# *In vivo* ^18^F-flortaucipir PET does not accurately support the staging of progressive supranuclear palsy

**DOI:** 10.2967/jnumed.121.262985

**Published:** 2021-11-18

**Authors:** Maura Malpetti, Sanne S. Kaalund, Kamen A. Tsvetanov, Timothy Rittman, Mayen Briggs, Kieren S.J. Allinson, Luca Passamonti, Negin Holland, P. Simon Jones, Tim D. Fryer, Young T. Hong, Antonina Kouli, W. Richard Bevan-Jones, Elijah Mak, George Savulich, Maria Grazia Spillantini, Franklin I. Aigbirhio, Caroline H. Williams-Gray, John T. O’Brien, James B. Rowe

**Affiliations:** 1 Department of Clinical Neurosciences, University of Cambridge, Cambridge, UK; 2 Cambridge University Hospitals NHS Foundation Trust, Cambridge, UK; 3 Cambridge University Brain Bank, Cambridge, UK; 4 Istituto di Bioimmagini e Fisiologia Molecolare (IBFM), Consiglio Nazionale delle Ricerche (CNR), Milano, Italy; 5 Wolfson Brain Imaging Centre, University of Cambridge; 6 Department of Psychiatry, University of Cambridge, Cambridge, UK; 7 Medical Research Council Cognition and Brain Sciences Unit, University of Cambridge, UK

**Keywords:** Progressive Supranuclear Palsy, ^18^F-flortaucipir, staging, tau pathology, PET-to-autopsy

## Abstract

Progressive Supranuclear Palsy (PSP) is a neurodegenerative disorder characterised by neuro-glial tau pathology. A new staging system for PSP pathology at *post-mortem* has been described and validated. We used a data-driven approach to test whether *post-mortem* pathological staging in PSP can be reproduced *in vivo* with ^18^F-flortaucipir PET.

**Methods:**

N=42 patients with probable PSP and N=39 controls underwent ^18^F-flortaucipir PET. Conditional inference tree analyses on regional binding potential values identified absent/present pathology thresholds to define *in vivo* staging. Following the staging system for PSP pathology, the combination of absent/present values across all regions was evaluated to assign each participant to in vivo stages. Analysis of variance was applied to analyse differences among means of disease severity between stages. *In vivo* staging was compared with *post-mortem* staging in N=9 patients who also had *post-mortem* confirmation of the diagnosis and stage.

**Results:**

Stage assignment was estimable in 41 patients: N=10 patients were classified in stage I/II, N=26 in stage III/IV, N=5 in stage V/VI, while N=1 was not classifiable. An explorative sub-staging identified N=2 patients in stage I, N=8 in stage II, N=9 in stage III, N=17 in stage IV and N=5 in stage V. However, the nominal ^18^F-flortaucipir derived stage was not associated with clinical severity and was not indicative of pathology staging at *post-mortem*.

**Conclusion:**

^18^F-flortaucipir PET *in vivo* does not correspond to neuropathological staging in PSP. This analytic approach, seeking to mirror *in vivo* the neuropathology staging with PET-to-autopsy correlational analyses might enable *in vivo* staging with next-generation PET tracers for tau, but further evidence and comparison with *post-mortem* data are needed.

## Graphical abstract

**Graphical abstract:**
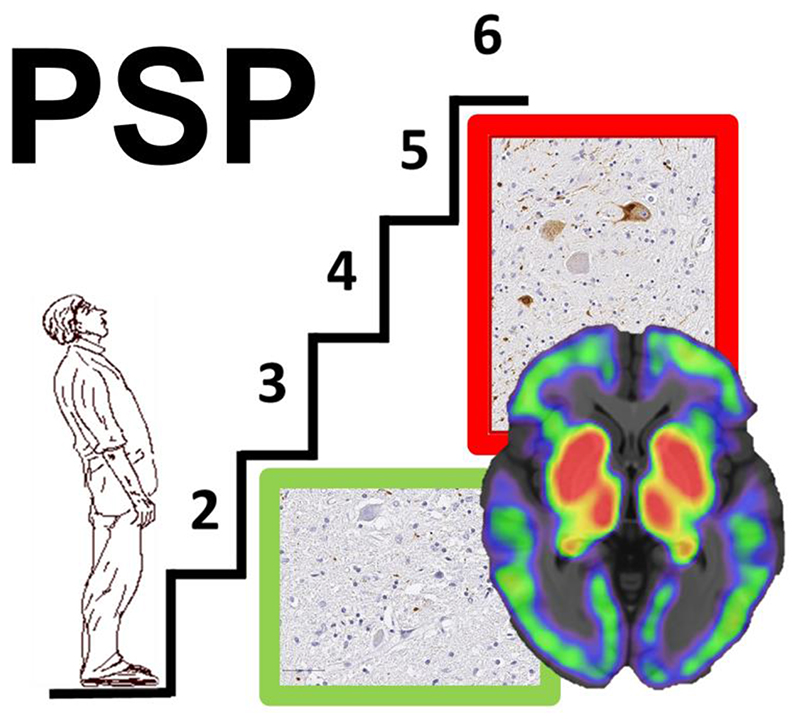

## Introduction

Progressive supranuclear palsy (PSP) is a severe neurodegenerative disorder resulting in diverse clinical phenotypes with restricted eye movements, akinetic-rigidity, falls, cognitive and behavioural deficits (1). The neuropathology of PSP is characterised by intracellular aggregates of 4-repeat (4R) tau in neurons and glia (2–5), which are distributed in a progressive sequence starting in the substantia nigra, globus pallidus and subthalamic nucleus, then the precentral gyrus in the cerebral cortex, pons and striatum, before reaching the cerebellum and/or frontal cortex (6). Later, the neuroglial pathology may extend to the occipital cortex (7).

A new neuropathological staging system has recently been introduced, and independently validated, for PSP tau pathology at *post-mortem* (7,8). This method confirms an association between pathology stage and clinical severity prior to death. To stage disease severity *ante mortem* requires a different methodology. For the tauopathy of Alzheimer’s disease for example, ^18^F-flortaucipir positron emission tomography (^18^F-flortaucipir PET) can reproduce the staging *in vivo* (9–16).

Here, we test whether regional binding of the radioligand ^18^F-flortaucipir (also known as ^18^F-AV-1451) quantified using non-displaceable binding potential can be used to replicate the staging of PSP pathology *in vivo*. We validate the staging in two ways: (i) the correlation with clinical severity at the time of ^18^F-flortaucipir PET; and (ii) neuropathological staging of a subset of participants *post-mortem*.

## Materials And Methods

### Participants

We recruited N=42 patients with a clinical diagnosis of probable PSP using MDS-PSP 2017 criteria (1) (female/male: 19/23; age: 70.3 ± 7.0 [50-84]; N=35 PSP Richardson’s syndrome and N=7 other phenotypes), and included data from N=39 cognitively healthy controls (female/male: 16/23; age: 65.8 ± 8.2 [48-84]; Addenbrooke’s Cognitive Examination (ACE-R/ACE-III): 96.2 ± 2.9 [89-100]). Disease severity was measured using the PSP rating scale (PSPRS: 36.6 ± 14.2 [10-74]). Nine out of 42 patients have to date donated their brains to the Cambridge Brain Bank, after a mean of 2.45 (± 0.98) years from PET. All these patients had *post-mortem* pathological confirmation of PSP pathology.

All participants underwent dynamic PET imaging for 90 minutes following ^18^F-flortaucipir injection (patients: N=22 GE Signa PET/MR, N=13 GE Discovery 690 PET/CT, N=7 GE Advance PET; controls: N=24 GE Signa PET/MR, N=7 GE Discovery 690 PET/CT, N=8 GE Advance PET; all scanners GE Healthcare, Waukesha, USA). The sensitivity advantage of the PET/MR scanner was used to reduce the target injection activity by 50% compared to the PET and PET/CT scans, leading to a comparable signal-to-noise ratio in the acquired data across the scanners. Full details of the imaging protocols have been published elsewhere (17,18). Seven out of 9 patients who donated their brains underwent ^18^F-flortaucipir imaging with the GE Discovery 690 PET/CT, with the other two scanned with the GE Advance PET.

Relevant approvals were granted by the Cambridge Research Ethics Committee (references: 13/EE/0104, 16/EE/0529, 18/EE/0059), the East of England - Essex Research Ethics Committee (16/EE/0445), and the Administration of Radioactive Substances Advisory Committee. All participants provided written informed consent in accordance with the Declaration of Helsinki.

### Determination Of Regional ^18^F-flortaucipir Binding

^18^F-flortaucipir non-displaceable binding potential was calculated in regions of interest corresponding closely to those used for *post-mortem* staging of PSP by Kovacs et al: globus pallidus, cerebellum (white matter and dentate nucleus), middle frontal gyrus and occipital lobe (lingual gyrus and cuneus) (Supplemental Figure 1A). The striatum and subthalamic nucleus were excluded because of ^18^F-flortaucipir off-target binding and/or challenges in defining PET signal. Regional values were quantified using a modified version of the n30r83 Hammersmith atlas (www.brain-development.org), which includes parcellation of the brainstem and cerebellum, and a basis function implementation of the simplified reference tissue model (19), with cerebellar cortex grey matter as the reference region. Prior to kinetic modelling, regional PET data were corrected for partial volume effects from cerebrospinal fluid by dividing by the mean regional grey-matter plus white-matter fraction determined from SPM segmentation. Left and right regional non-displaceable binding potential values were averaged bilaterally. Using regional mean and standard deviation (SD) values from controls, w-scores were calculated (Supplemental Figure 1B), accounting for phenotypic and systematic differences, such as age and scanner type (PET/MR vs. non-PET/MR); see Malpetti et al. (17) for a discussion on harmonisation of PET and PET/CT data.

### *In Vivo* Staging Based On ^18^F-flortaucipir Binding

#### Data-driven severity thresholds

To quantify pathology severity in each region, we used a conditional inference tree analysis to define in a data-driven manner region-specific ^18^F-flortaucipirbinding thresholds of w-scores, entering both patients and controls in the model. This method is similar to that used previously for imaging-based pseudo-Braak staging of Alzheimer’s disease (9). Specifically, region-specific thresholds were identified using a nonparametric binary recursive partitioning with the function “*ctree*” in R (v. 4.0.0), and running this tree-analysis on w-scores for each region separately. Using these region-specific thresholds, binary severity scores were assigned to individual regional w-scores (w-score ≤ regional threshold: 0 or absent; w-score > regional threshold: 1 or present).

#### *In vivo* staging

**Figure 1:**
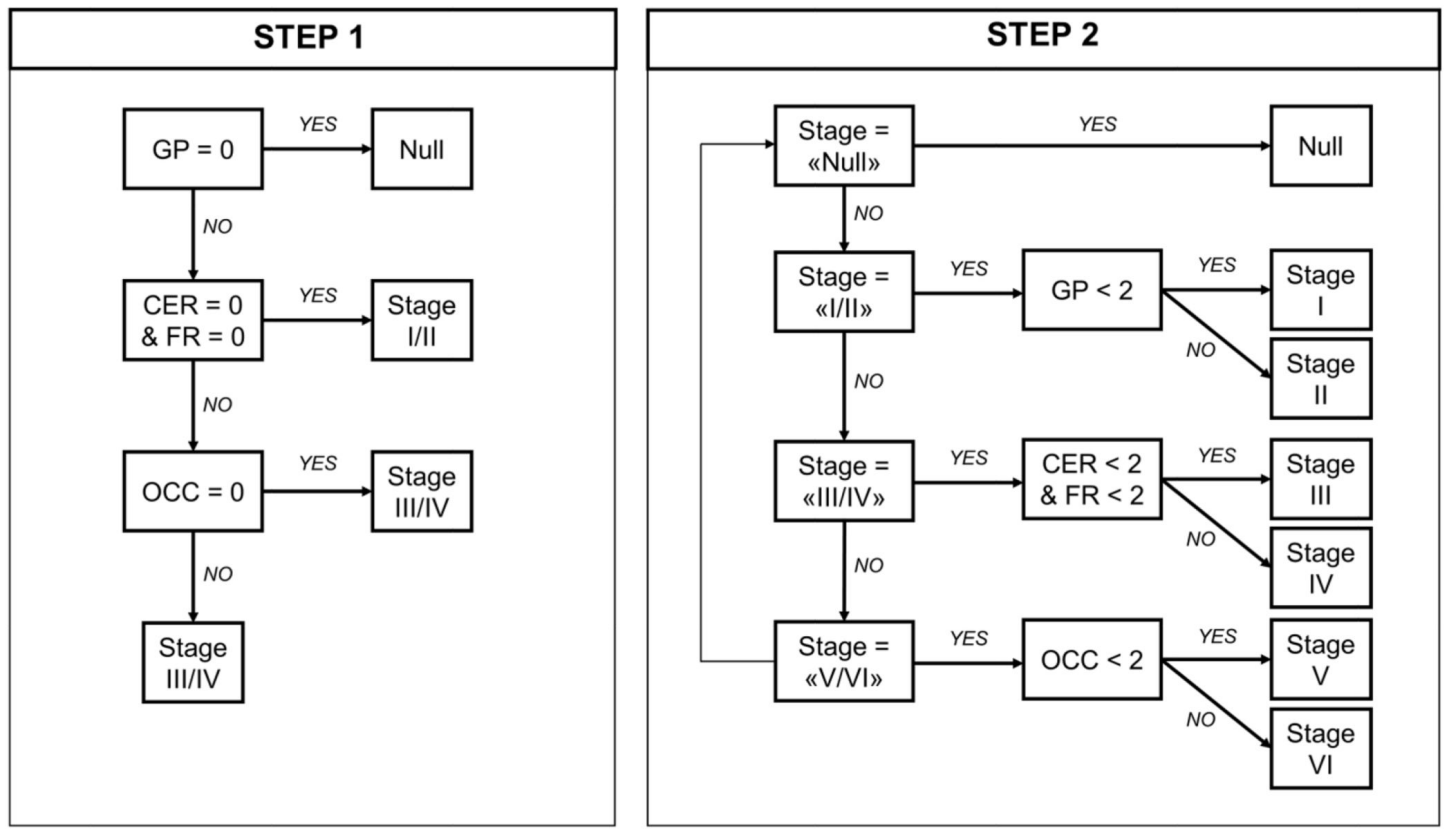
*In vivo* staging if-else rules. Step 1: *in vivo* stages are defined with cumulative evidence of absence (region = 0) or presence (region = 1) of pathology in each of the five regions considered, as defined by region-specific thresholds (regional w-score > threshold = 1; regional w-score ≤ threshold = 0). Step 2: *in vivo* sub-stages are defined within each step-1 stage considering a 3-level pathology severity scale (0 = none; 1 = mild/moderate pathology; 2 = moderate/severe pathology). Regions: globus pallidus (GP), cerebellum (CER, white matter and dentate nucleus), middle frontal gyrus (FR) and occipital lobe (OCC – lingual gyrus and cuneus).

*First,* following the staging system described by Kovacs et al. (7), which is based on cumulative and progressive pathology severity, the combination of absent/present values across all 4 regions was evaluated to assign each participant to Stages I/II, III/IV or V/VI (Figure 1, “STEP 1”). *Second,* as explorative analysis, within each stage defined in the previous step a 3-point pathology severity system was applied to each region (w-score ≤ regional threshold: absent, coded as 0; w-score > regional threshold: mild/moderate pathology, coded as 1; w-score > 2*threshold: moderate/severe pathology, coded as 2) and one of the six stages were assigned accordingly (Stage I-VI; Figure 1, “STEP 2”). We repeated these staging analyses with a second analytical approach, using a pre-selected number of SD from region-specific non-displaceable binding potential control means to define pathology severity (Supplemental Material & Supplemental Figure 2). Analysis of variance (ANOVA) was applied to analyse differences among means of disease severity (PSPRS) between stages.

### *Post-Mortem* Diagnosis And Staging Based On Immunohistochemistry

Tissue blocks from the left hemisphere were sampled according to NINDS standard guidance for neurodegenerative diseases from brainstem, subcortical and cortical areas and were evaluated for the initial pathological diagnosis of PSP (hyperphosphorylated tau; AT8, MN1020, Thermo Scientific, USA) and possible concomitant pathologies of amyloid beta (Clone 6F/3D, M0872, Dako, Denmark), alpha-synuclein (SA3400, Enzo life sciences, USA), TDP-43 (TIP-PTD-P02, Cosmo Bio Co LTD, Japan), and vascular pathology. Following the staging scheme previously described (7,8), we evaluated neuronal and oligodendroglia tau-pathology in the globus pallidus, subthalamic nucleus, and cerebellar white matter and dentate nucleus, and astrocytic tau-pathology in the striatum, middle frontal gyrus, and occipital cortex. The regional cytopathologies were rated on a 4-level system (none, mild, moderate and severe) using the guidelines proposed in Briggs et al. (2021). *In vivo* staging results with both data-driven and standard-deviation approaches were compared with *post-mortem* staging in these 9 patients.

## Results

**Figure 2:**
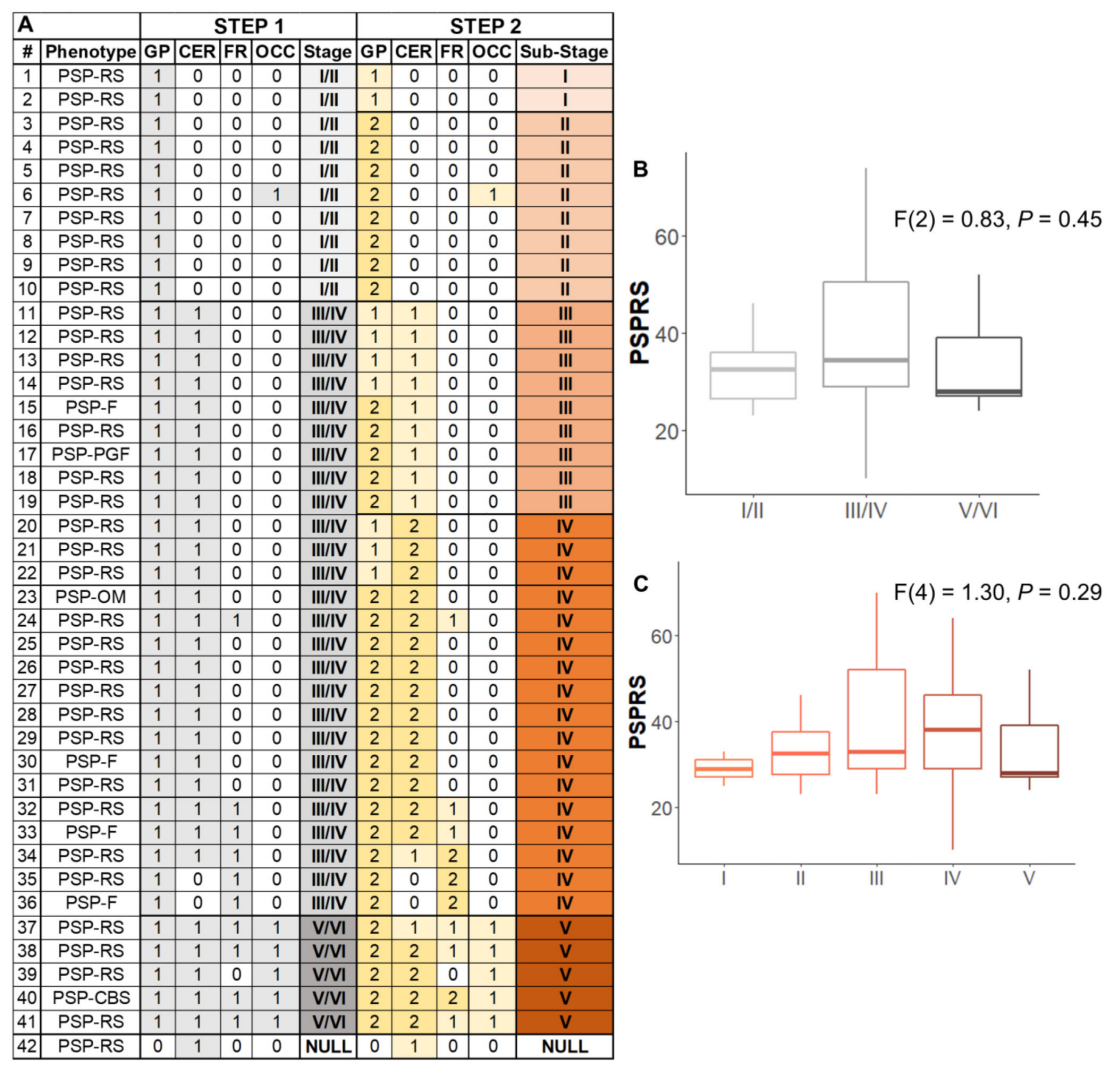
*In vivo* staging based on data-driven thresholds. Panel A: severity scores are reported for each group of regions considered to define *in vivo* stages (STEP 1: 0 = absent 1 = present) and sub-stages (STEP 2: 0 = none; 1 = mild/moderate pathology; 2 = moderate/severe pathology). Abbreviations: progressive supranuclear palsy (PSP), PSP-Richardson’s syndrome (-RS), PSP-frontal (-F), PSP-progressive gait freezing (-PGF), PSP-oculomotor (-OM), PSP-corticobasal syndrome (-CBS), globus pallidus (GP), cerebellum (CER, white matter and dentate nucleus), middle frontal gyrus (FR) and occipital lobe (OCC – lingual gyrus and cuneus). Panel B and C: boxplots of PSP rating scale (PSPRS) scores by stages defined with STEP 1 (panel B) and STEP 2 (panel C).

The conditional inference tree analysis identified region-specific pathological thresholds of ^18^F-flortaucipir binding for globus pallidus (w-score > 0.795), cerebellum white matter (w-score > 0.783) and dentate nucleus (w-score > 0.845), and middle frontal gyrus (w-score > 1.416). For the occipital lobe, the analysis did not identify the threshold, so we used 1.645 as the w-score critical value (p=0.05). A simple set of decision rules (Figure 1) enabled plausible Kovacs stages to be estimated in 41 patients (Figure 2A): N=10 patients were classified in stage I/II because of increased ^18^F-flortaucipir binding limited to globus pallidus; N=26 in stage III/IV with additional increased ^18^F-flortaucipir binding in frontal and/or cerebellum regions; N=5 in stage V/VI with additional increased ^18^F-flortaucipir binding in occipital lobe; while N=1 was not classifiable as no increased binding in globus pallidus was found. The explorative sub-staging (6 stages) identified N=2 patients in stage I (mild/moderate pathology in globus pallidus), N=8 in stage II (moderate/severe pathology in globus pallidus), N=9 in stage III (mild/moderate in frontal lobe and/or cerebellum), N=17 in stage IV (moderate/severe in frontal lobe and/or cerebellum) and N=5 in stage V (mild/moderate in occipital lobe). Applying the same approach to controls, N=31 participants were classified in no stage, N=5 in stage I, N=1 in stage II and N=2 in stage III. Four patients (Figure 2A, patients no: 6,35,36,39) showed an atypical severity pattern that was discordant with the description of Kovacs et al.

**Figure 3:**
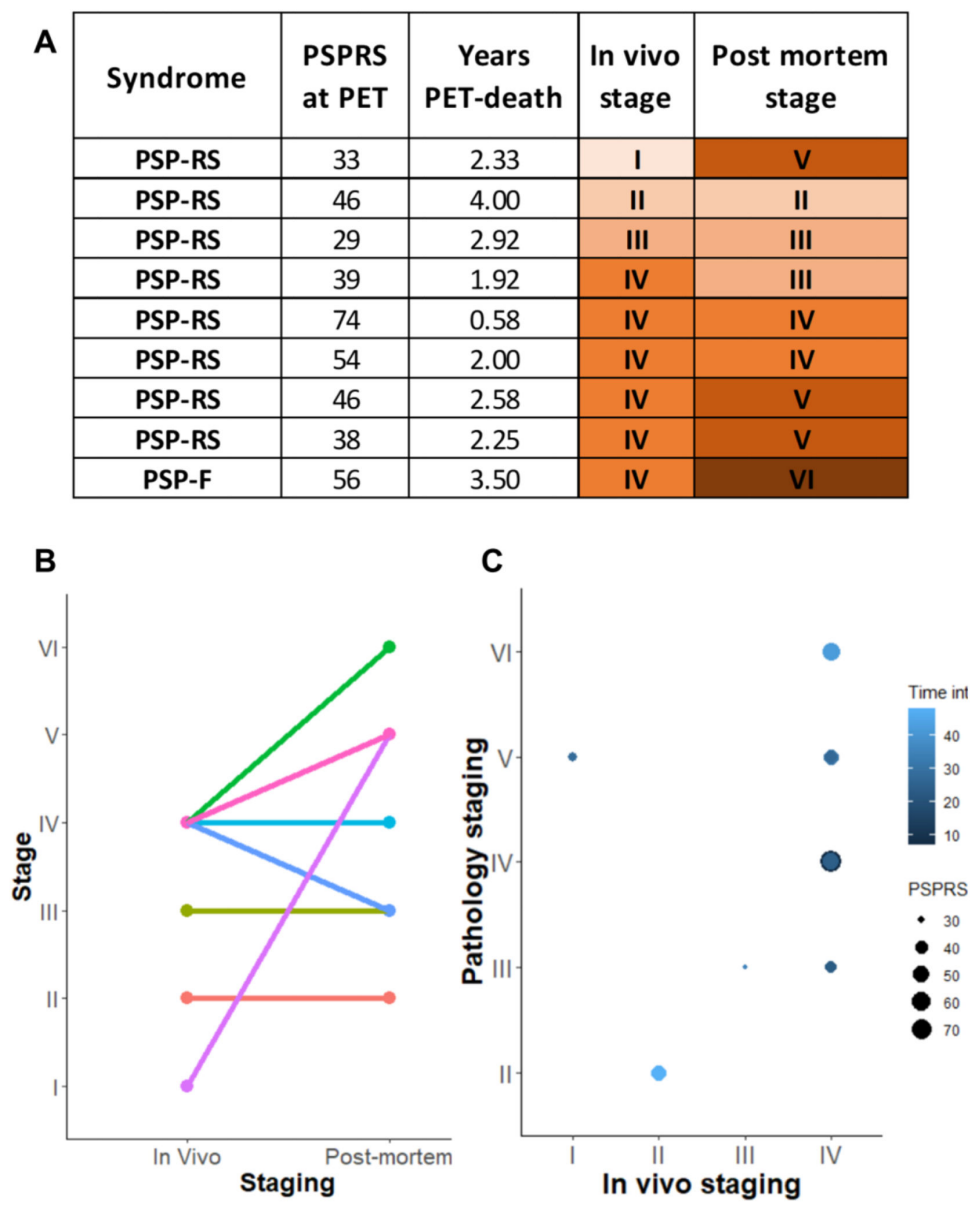
Comparison between *in vivo* and *post-mortem* stages for 9 patients who underwent ^18^F-flortaucipir PET and pathology autopsy. Panel A: clinical and staging details; panel B: single subject (lines) comparisons between *in vivo* and *post-mortem* staging; panel C: graphical representation of PET-to-death time interval and clinical severity on the association between *in vivo* and *post-mortem* staging. Abbreviations: progressive supranuclear palsy (PSP), PSP-Richardson’s syndrome (-RS), PSP-frontal (-F), PSP rating scale (PSPRS), PET-death time interval (Time int).

**Figure 4:**
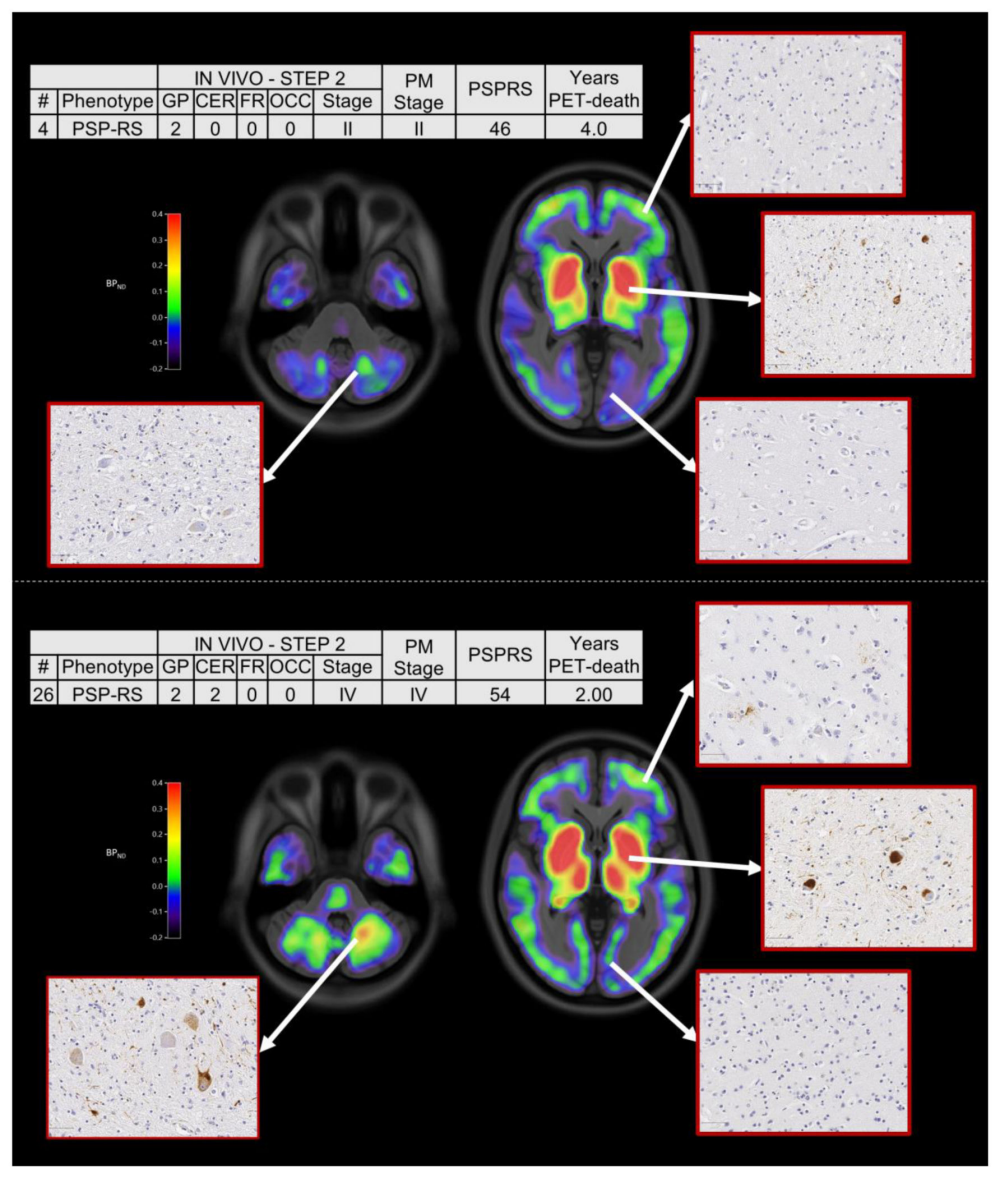
^18^F-flortaucipir non-displaceable binding potential (BP_ND_) maps and *post-mortem* staining, and related clinical details, for two patients classified into Stage II (top panel) and Stage IV (bottom panel) with both *in vivo* and *post-mortem* staging. The spatially normalised BPND maps are shown in radiological format overlaid on the ICBM MNI152 2009a T1 MRI template. Abbreviations: progressive supranuclear palsy (PSP), PSP-Richardson’s syndrome (-RS), globus pallidus (GP), cerebellum (CER), middle frontal gyrus (FR), occipital lobe (OCC), post-mortem stage (PM stage), PSP rating scale (PSPRS).

Across all patients, the estimated *in vivo* stages did not relate to clinical severity (ANOVA p>0.05, Figure 2B and Figure 2C). In 8 of the 9 patients who donated their brains, pathology stage as determined by *in vivo* ^18^F-flortaucipir PET, was less than or equal to that determined at *post-mortem* (Figure 3). *In vivo* and *post-mortem* staging were not significantly correlated (Spearman’s r = 0.168, p = 0.67). Correlation analyses were also tested on the residuals of each staging variable (*in vivo* and *post-mortem* staging) after regressing out clinical severity (PSPRS scores) and PET-death time interval. The correlation was not statistically significant (Spearman’s r = 0.150, p = 0.70). Figure 4 gives examples of ^18^F-flortaucipir non-displaceable binding potential maps and corresponding *post-mortem* staining data for patients who were classified into stage II (patient no. 4) and stage IV (patient no. 26) with both *in vivo* and *post-mortem* staging.

## Discussion

The principal finding of this study is that ^18^F-flortaucipir PET does not provide accurate *in vivo* staging in PSP corresponding to the neuropathological staging. The nominal stage derived from ^18^F-flortaucipir PET did not correlate with disease severity, nor relate to the staging *post-mortem*.

As a result of the data-driven *in vivo* staging system, compared to controls, we observed higher ^18^F-flortaucipir binding in all but one patient in globus pallidus, with a few patients showing increased ^18^F-FTP binding in occipital cortex (Figure 2A). This regional distribution of ^18^F-flortaucipir binding is in line with the pathological description of PSP and what has previously been described for ^18^F-flortaucipir in PSP (13,17,18,20). Whereas the ^18^F-flortaucipir binding patterns allowed us to nominally apply the PSP pathology staging *in vivo*, the *in vivo* staging was not systematically predictive of pathology staging at *post-mortem*. As expected because of the time interval between PET scan and autopsy, in 8 out of 9 cases with autopsy, the individual *in vivo* staging was less than or equal to the *post-mortem* staging. However, four patients who were labelled as Stage IV *in vivo*, were then classified in 4 different stages at *post-mortem* (Figure 3). Neither clinical severity, nor the time interval between PET scan and death were useful for predicting the individual *post-mortem* stage from the *in vivo* staging.

The number of patients with a positive signal for ^18^F-flortaucipir in the cerebellum, N = 29, exceeded the number of patients positive for frontal ^18^F-flortaucipir binding, N = 10. While this may reflect earlier involvement of the cerebellum in our cohort, regional differences in the density of tau aggregates and predominant cytopathologies could contribute to regional differences in tracer retention (11,13,21), e.g. neuronal and oligodendroglial tau predominates in the cerebellum while astrocytic tau predominates in cortical regions.

Off-target binding is well-characterised for ^18^F-flortaucipir, but this problem alone would still leave open the utility to quantify tau pathology in areas without significant mono-amine oxidase levels or neuromelanin, such as cerebellum and medial frontal cerebral cortex (22). However, recent PET-to-autopsy correlational studies suggested that ^18^F-flortaucipir PET does not reliably correspond to *post-mortem* tau pathology in non-Alzheimer’s tauopathies (13,23). This suggests that ^18^F-flortaucipir lacks sensitivity in non-Alzheimer tau pathology. This may explain the underperformance of this tracer in defining an *in vivo* classification that systematically aligns with *post-mortem* staging. Next-generation tau tracers may prove to be more useful to track *in vivo* PSP pathology progression because of a combination between good affinity for 4R tau and lower off-target binding to monoamine oxidases (i.e. ^18^F-PI-2620 (24)). However, evidence from PET-to-autopsy studies is needed for these new ligands, together with better segmentation and signal detection from small regions. This would be particularly important for early-stage pathology detection, and the classification of Stage I/II of the Kovacs et al system.

## Conclusion

We conclude that ^18^F-flortaucipir PET is not a useful marker of neuropathological stage in PSP, despite increased binding and some regional concordance between tau pathology and ligand binding. This analytical approach, seeking to mirror *in vivo* the neuropathology staging with PET-to-autopsy correlational analyses, could be applied to test next-generation tau PET tracers. However, comparisons with *post-mortem* data are also required.

## Supplementary Material

Supplemental Materials

## Key Points

### Question

can the novel *post-mortem* pathological staging in Progressive Supranuclear Palsy (PSP) be reproduced *in vivo* with ^18^F-flortaucipir PET?

### Pertinent findings

Conditional inference tree analyses were performed on regional ^18^F-flortaucipir PET binding potential values to define *in vivo* staging in 42 patients with probable PSP, comparing the results in 9 participants with *post-mortem* confirmation of the diagnosis and stage. ^18^F-flortaucipir PET does not provide accurate *in vivo* staging in PSP. In particular, the nominal stage derived from ^18^F-flortaucipir PET did not correlate with disease severity, nor relate to the staging *post-mortem*.

### Implications for patient care

This analytic approach, seeking to mirror *in vivo* the neuropathology staging with PET-to-autopsy correlational analyses, may be more effective with next-generation PET tracers for tau.
