# In Vivo ^18^F-Flortaucipir PET Does Not Accurately Support the Staging of Progressive Supranuclear Palsy

**DOI:** 10.2967/jnumed.121.262985

**Published:** 2022-07

**Authors:** Maura Malpetti, Sanne S. Kaalund, Kamen A. Tsvetanov, Timothy Rittman, Mayen Briggs, Kieren S.J. Allinson, Luca Passamonti, Negin Holland, P. Simon Jones, Tim D. Fryer, Young T. Hong, Antonina Kouli, W. Richard Bevan-Jones, Elijah Mak, George Savulich, Maria Grazia Spillantini, Franklin I. Aigbirhio, Caroline H. Williams-Gray, John T. O’Brien, James B. Rowe

**Affiliations:** 1 Department of Clinical Neurosciences, University of Cambridge, Cambridge, United Kingdom;; 2 Cambridge University Hospitals NHS Foundation Trust, Cambridge, United Kingdom;; 3 Cambridge University Brain Bank, Cambridge, United Kingdom;; 4 Istituto di Bioimmagini e Fisiologia Molecolare (IBFM), Consiglio Nazionale delle Ricerche (CNR), Milano, Italy;; 5 Wolfson Brain Imaging Centre, University of Cambridge, Cambridge, United Kingdom;; 6 Department of Psychiatry, University of Cambridge, Cambridge, United Kingdom; and; 7 Medical Research Council Cognition and Brain Sciences Unit, University of Cambridge, Cambridge, United Kingdom

**Keywords:** progressive supranuclear palsy, ^18^F-flortaucipir, staging, tau pathology, PET-to-autopsy studies

## Abstract

Progressive supranuclear palsy (PSP) is a neurodegenerative disorder characterized by neuroglial tau pathology. A new staging system for PSP pathology postmortem has been described and validated. We used a data-driven approach to test whether postmortem pathologic staging in PSP can be reproduced in vivo with ^18^F-flortaucipir PET. **Methods:** Forty-two patients with probable PSP and 39 controls underwent ^18^F-flortaucipir PET. Conditional inference tree analyses on regional binding potential values identified absent/present pathology thresholds to define in vivo staging. Following the postmortem staging approach for PSP pathology, we evaluated the combinations of absent/present pathology (or abnormal/normal PET signal) across all regions to assign each participant to in vivo stages. ANOVA was applied to analyze differences among means of disease severity between stages. In vivo staging was compared with postmortem staging in 9 patients who also had postmortem confirmation of the diagnosis and stage. **Results:** Stage assignment was estimable in 41 patients: 10, 26, and 5 patients were classified in stage I/II, stage III/IV, and stage V/VI, respectively, whereas 1 patient was not classifiable. Explorative substaging identified 2 patients in stage I, 8 in stage II, 9 in stage III, 17 in stage IV, and 5 in stage V. However, the nominal ^18^F-flortaucipir--derived stage was not associated with clinical severity and was not indicative of pathology staging postmortem. **Conclusion:** ^18^F-flortaucipir PET in vivo does not correspond to neuropathologic staging in PSP. This analytic approach, seeking to mirror in vivo neuropathology staging with PET-to-autopsy correlational analyses, might enable in vivo staging with next-generation tau PET tracers; however, further evidence and comparisons with postmortem data are needed.

## Visual Abstract

**Figure:**
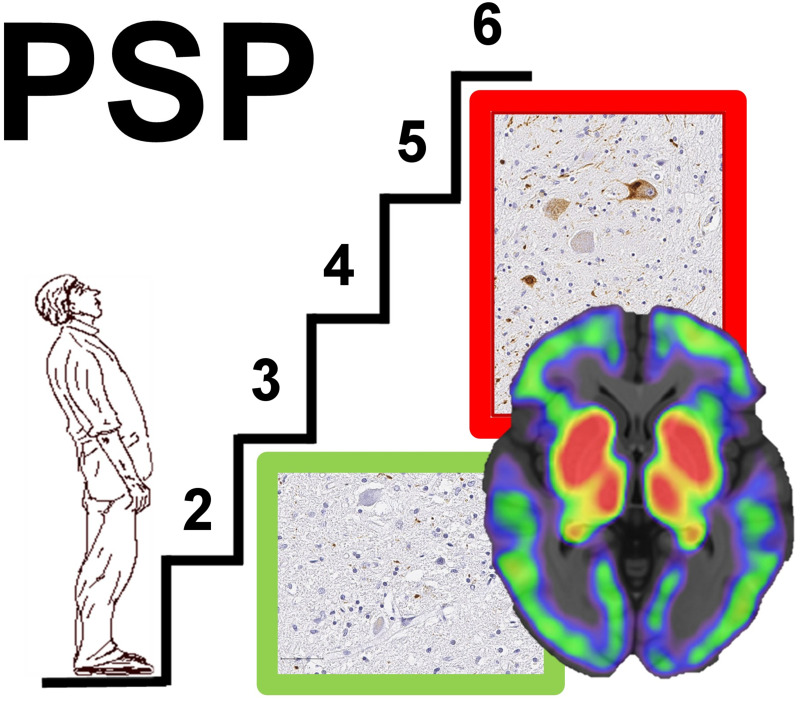

Progressive supranuclear palsy (PSP) is a severe neurodegenerative disorder resulting in diverse clinical phenotypes with restricted eye movements, an akinetic–rigid syndrome, falls, and cognitive and behavioral deficits (*1*). The neuropathology of PSP is characterized by intracellular aggregates of 4-repeat tau in neurons and glia (*2*–*5*); these aggregates are distributed in a progressive sequence starting in the substantia nigra, globus pallidus and subthalamic nucleus, then pons, striatum and the precentral gyrus in the cerebral cortex, before reaching the cerebellum and frontal cortex (*6*). Later, the neuroglial pathology might extend to the occipital cortex (*7*).

A new neuropathologic staging system for PSP tau pathology postmortem was recently introduced and independently validated (*7**,**8*). This method confirms an association between pathology stage and clinical severity before death. To stage disease severity antemortem requires a different methodology. For the tauopathy of Alzheimer disease, for example, ^18^F-flortaucipir PET can reproduce staging in vivo (*9*–*16*).

Here, we test whether regional binding of the radioligand ^18^F-flortaucipir (also known as ^18^F-AV-1451), quantified using nondisplaceable binding potential, can be used to replicate the staging of PSP pathology in vivo. We validated the staging in 2 ways: correlation with clinical severity at the time of ^18^F-flortaucipir PET and neuropathologic staging of a subset of participants postmortem.

## MATERIALS AND METHODS

### Participants

We recruited 42 patients with a clinical diagnosis of probable PSP using Movement Disorder Society PSP 2017 criteria (*1*) (19 women and 23 men; mean age, 70.3 y [SD, 7.0 y; range, 50–84 y]; 35 with PSP Richardson syndrome and 7 with other phenotypes) and included data from 39 cognitively healthy controls (16 women and 23 men; mean age, 65.8 y [SD, 8.2 y; range, 48–84 y]; mean revised Addenbrooke’s Cognitive Examination score, 96.2 [SD, 2.9; range, 89–100]). Disease severity was measured using the PSP rating scale (PSPRS) (mean, 36.6 [SD, 14.2; range, 10–74]). To date, 9 of the 42 patients donated their brain to the Cambridge Brain Bank, after a mean of 2.45 (SD, 0.98) years from PET. All of these patients had postmortem pathologic confirmation of PSP pathology.

All participants underwent dynamic PET imaging for 90 min after ^18^F-flortaucipir injection (GE Signa PET/MRI for 22 patients; GE Discovery 690 PET/CT for 13 patients; GE Advance PET for 7 patients; GE Signa PET/MRI for 24 controls; GE Discovery 690 PET/CT for 7 controls; GE Advance PET for 8 controls) (all scanners were from GE Healthcare). The sensitivity advantage of the PET/MRI scanner was used to reduce the target injection activity by 50% compared with that used in the PET and PET/CT scans, leading to a comparable signal-to-noise ratio in the acquired data across the scanners. Full details of the imaging protocols were published elsewhere (*17**,**18*). Seven of the 9 patients who donated their brains underwent ^18^F-flortaucipir imaging with GE Discovery 690 PET/CT; the other 2 were scanned with GE Advance PET.

Relevant approvals were granted by the Cambridge Research Ethics Committee (references: 13/EE/0104, 16/EE/0529, and 18/EE/0059), the East of England–Essex Research Ethics Committee (16/EE/0445), and the Administration of Radioactive Substances Advisory Committee. All participants provided written informed consent in accordance with the Declaration of Helsinki.

### Determination of Regional ^18^F-Flortaucipir Binding

^18^F-flortaucipir nondisplaceable binding potential was calculated in regions of interest corresponding closely to those used for postmortem staging of PSP by Kovacs et al. (*7*): globus pallidus, cerebellum (white matter and dentate nucleus), middle frontal gyrus, and occipital lobe (lingual gyrus and cuneus) (Supplemental Fig. 1A) (supplemental materials are available at http://jnm.snmjournals.org). The striatum and subthalamic nucleus were excluded because of ^18^F-flortaucipir off-target binding or challenges in defining the PET signal. Regional values were quantified using a modified version of the n30r83 Hammersmith atlas (http://brain-development.org/brain-atlases/adult-brain-atlases/adult-brain-maximum-probability-map-hammers-mith-atlas-n30r83-in-mni-space/), which includes parcellation of the brain stem and cerebellum, and a basis function implementation of the simplified reference tissue model (*19*), with cerebellar cortex gray matter as the reference region. Before kinetic modeling, regional PET data were corrected for partial-volume effects from cerebrospinal fluid by dividing the regional PET value by the mean regional gray matter plus white matter fraction determined from Statistical Parametric Mapping (SPM12, https://www.fil.ion.ucl.ac.uk/spm/) segmentation. Left and right regional nondisplaceable binding potential values were averaged bilaterally. Using regional mean and SD values from controls, we calculated w-scores (z-scores adjusted for the effect of covariates, Supplemental Fig. 1B), accounting for phenotypic and systematic differences, such as age and scanner type (PET/MRI vs. non-PET/MRI); see Malpetti et al. (*17*) for a discussion on harmonization of PET and PET/CT data.

### In Vivo Staging Based on ^18^F-Flortaucipir Binding

#### Data-Driven Severity Thresholds

To quantify pathology severity in each region, we used a conditional inference tree analysis to define in a data-driven manner region-specific ^18^F-flortaucipir binding thresholds of w-scores, entering both patients and controls in the model. This method is similar to that used previously for imaging-based pseudo-Braak staging of Alzheimer disease (*9*). Specifically, region-specific thresholds were identified using nonparametric binary recursive partitioning with the function “*ctree*” in R (v. 4.0.0, R Core Team - R Foundation for Statistical Computing) and running this tree analysis on w-scores for each region separately. Using these region-specific thresholds, we assigned binary severity scores to individual regional w-scores (w-score ≤ regional threshold: 0 or absent; w-score > regional threshold: 1 or present).

#### In Vivo Staging

**FIGURE 1.**
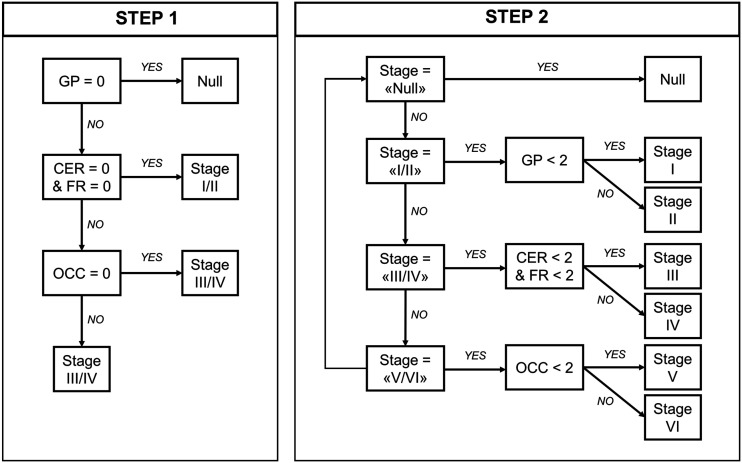
In vivo staging rules. Step 1: in vivo stages are defined with cumulative evidence of absence (region = 0) or presence (region = 1) of pathology in each of 5 regions considered, as defined by region-specific thresholds (regional w-score > threshold = 1; regional w-score ≤ threshold = 0). Step 2: in vivo substages are defined within each step 1 stage, considering 3-level pathology severity scale (0 = none; 1 = mild/moderate pathology; 2 = moderate/severe pathology). Regions: cerebellum (CER; white matter and dentate nucleus), middle frontal gyrus (FR), globus pallidus (GP), and occipital lobe (OCC; lingual gyrus and cuneus).

First, using the staging system described by Kovacs et al. (*7*), which is based on cumulative and progressive pathology severity, we evaluated the combination of absent/present values across all 4 regions to assign each participant to stages I/II, III/IV, or V/VI (step 1 on Fig. 1). Second*—*in an explorative analysis—within each stage defined in the previous step, a 3-point pathology severity system was applied to each region (w-score ≤ regional threshold: absent, coded as 0; w-score > regional threshold: mild/moderate pathology, coded as 1; w-score > 2 times the threshold: moderate/severe pathology, coded as 2), and 1 of the 6 stages was assigned accordingly (stages I–VI; step 2 on Fig. 1). We repeated these staging analyses with a second analytic approach, using a preselected number of SD values from region-specific nondisplaceable binding potential control means to define pathology severity (Supplemental Fig. 2). ANOVA was applied to analyze differences among means of disease severity (PSPRS) between stages.

**FIGURE 2.**
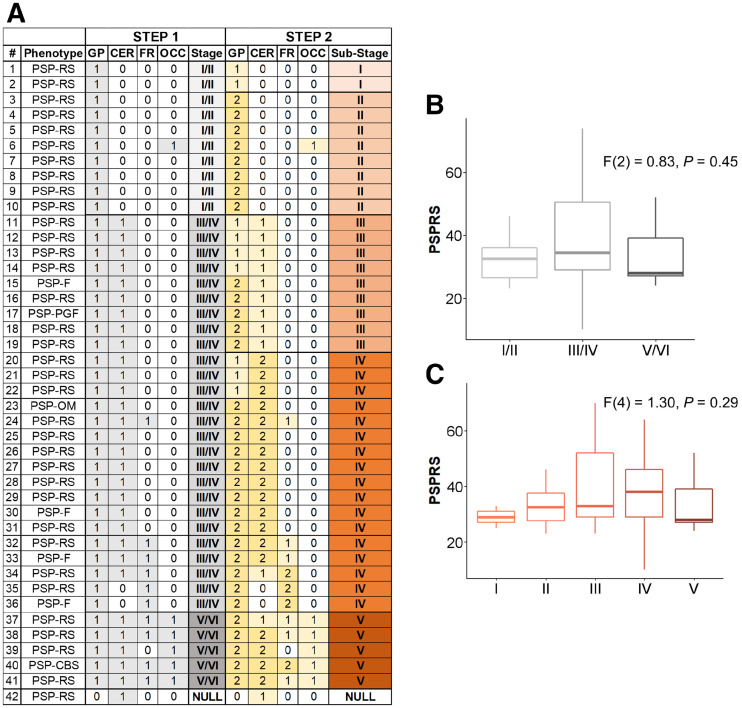
In vivo staging based on data-driven thresholds. (A) Severity scores are reported for each group of regions considered to define in vivo stages (step 1: 0 = absent; 1 = present) and substages (step 2: 0 = none; 1 = mild/moderate pathology; 2 = moderate/severe pathology). (B and C) Box plots of PSPRS scores by stages defined with step 1 (B) and step 2 (C). CER = cerebellum (white matter and dentate nucleus); FR = middle frontal gyrus; GP = globus pallidus; OCC = occipital lobe (lingual gyrus and cuneus); PSP-CBS = PSP-corticobasal syndrome; PSP-F = PSP-frontal; PSP-OM = PSP-oculomotor; PSP-PGF = PSP-progressive gait freezing; PSP-RS = PSP-Richardson syndrome.

### Postmortem Diagnosis and Staging Based on Immunohistochemistry

Tissue blocks of the left hemisphere were sampled according to National Institute of Neurological Disorders and Stroke standard guidance for neurodegenerative diseases from the brain stem, subcortical, and cortical areas. These were evaluated for the initial pathologic diagnosis of PSP (hyperphosphorylated tau; AT8, MN1020; Thermo Scientific, possible concomitant pathologies of amyloid β (clone 6F/3D, M0872; Dako), α-synuclein (SA3400; Enzo Life Sciences), and TDP-43 (TIP-PTD-P02; Cosmo Bio Co. Ltd.); and vascular pathology. Using the previously described staging scheme (*7**,**8*), we evaluated neuronal and oligodendroglial tau pathology in the globus pallidus, subthalamic nucleus, and cerebellar white matter and dentate nucleus and astrocytic tau pathology in the striatum, middle frontal gyrus, and occipital cortex. The regional cytopathologies were rated on a 4-level system (none, mild, moderate, and severe) using the guidelines proposed by Briggs et al. (*8*). In vivo staging results with both data-driven and SD approaches were compared with postmortem staging in the 9 patients who donated their brain.

## RESULTS

The conditional inference tree analysis identified region-specific pathologic thresholds of ^18^F-flortaucipir binding for the globus pallidus (w-score, >0.795), cerebellar white matter (w-score, >0.783) and dentate nucleus (w-score, >0.845), and middle frontal gyrus (w-score, >1.416). For the occipital lobe, the analysis did not identify the threshold, so we used 1.645 as the w-score critical value (*P* = 0.05). A simple set of decision rules (Fig. 1) enabled plausible Kovacs stages to be estimated in 41 patients (Fig. 2A): 10 patients were classified in stage I/II because of increased ^18^F-flortaucipir binding limited to the globus pallidus, 26 were classified in stage III/IV because of additional increased ^18^F-flortaucipir binding in the frontal or cerebellar regions, and 5 were classified in stage V/VI because of additional increased ^18^F-flortaucipir binding in the occipital lobe; 1 patient could not be classified because no increased binding was found in the globus pallidus. The explorative substaging (6 stages) identified 2 patients in stage I (mild/moderate pathology in the globus pallidus), 8 in stage II (moderate/severe pathology in the globus pallidus), 9 in stage III (mild/moderate pathology in the frontal lobe or cerebellum), 17 in stage IV (moderate/severe pathology in the frontal lobe or cerebellum), and 5 in stage V (mild/moderate pathology in the occipital lobe). When the same approach was applied to controls, 31, 5, 1, and 2 participants were classified in no stage, stage I, stage II, and stage III, respectively. Four patients (Fig. 2A, patients 6, 35, 36, and 39) showed an atypical severity pattern that was discordant with the description of Kovacs et al. (*7*).

**FIGURE 3.**
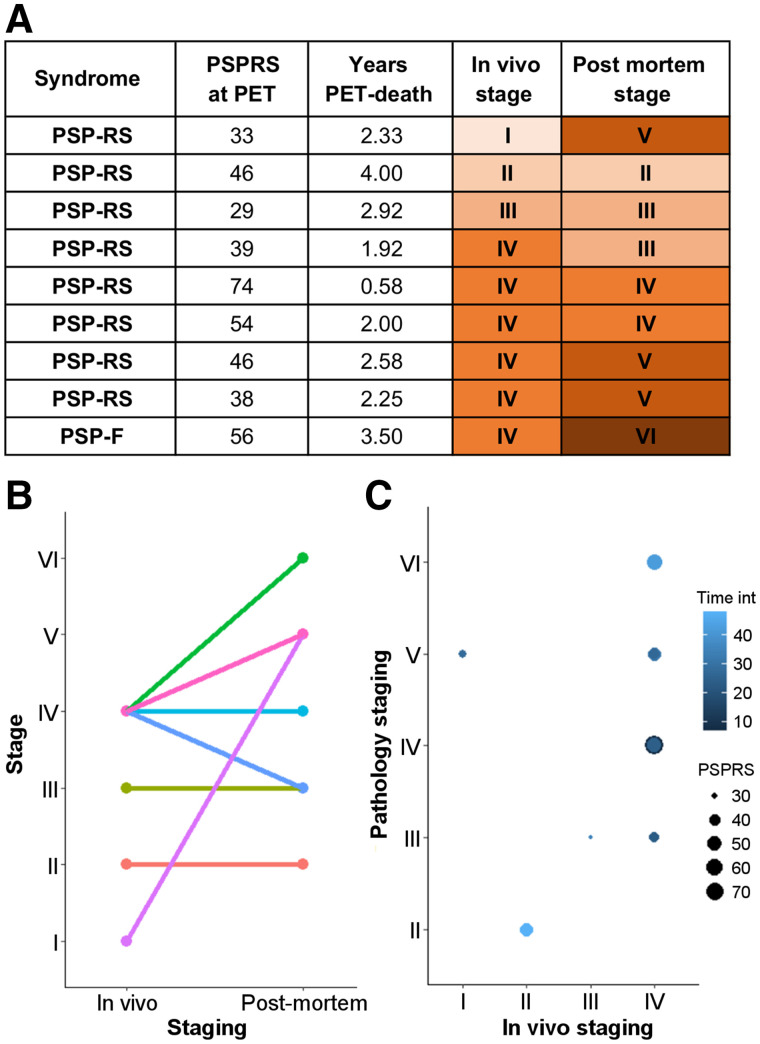
Comparison of in vivo and postmortem stages for 9 patients who underwent ^18^F-flortaucipir PET and pathology autopsy. (A) Clinical and staging details. (B) Single-subject (lines) comparisons of in vivo and postmortem staging. (C) Graphical representation of the effect of interval from PET to time of death (Time int) and clinical severity on association between in vivo staging and postmortem staging. PSP-F = PSP-frontal; PSP-RS = PSP-Richardson syndrome.

**FIGURE 4.**
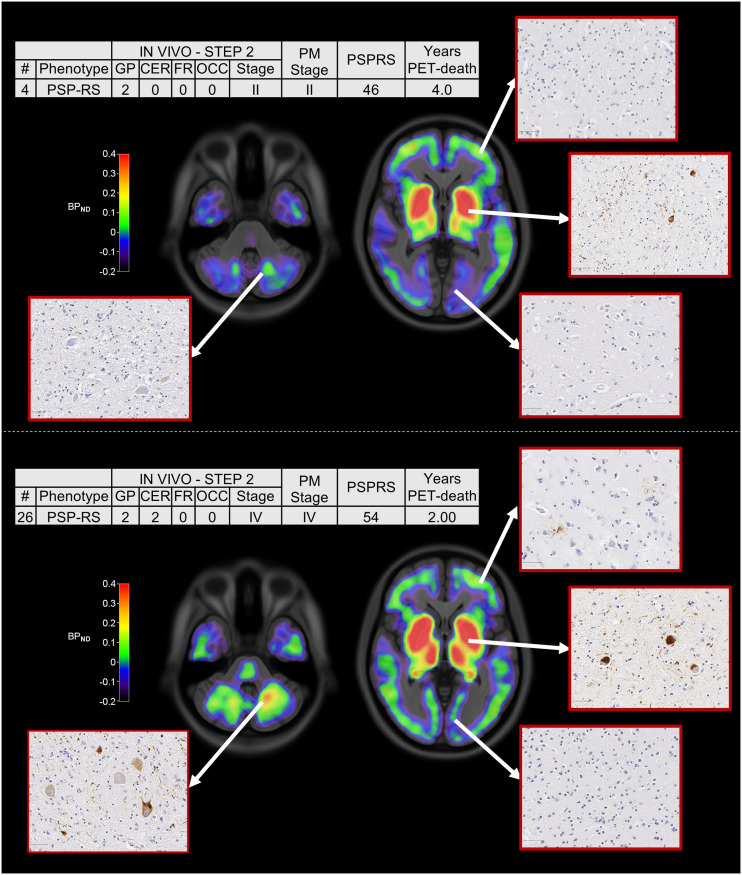
^18^F-flortaucipir nondisplaceable binding potential (BP_ND_) maps, postmortem staining, and related clinical details for 2 patients classified in stage II (top) and stage IV (bottom) with both in vivo staging and postmortem staging. Spatially normalized BP_ND_ maps are shown in radiologic format overlaid on ICBM MNI152 2009a T1 MRI template (https://www.bic.mni.mcgill.ca/ServicesAtlases/ICBM152NLin2009). CER = cerebellum; FR = middle frontal gyrus; GP = globus pallidus; OCC = occipital lobe; PM stage = postmortem stage; PSP-RS = PSP-Richardson syndrome.

Across all patients, the estimated in vivo stages did not relate to clinical severity (*P* > 0.05 in an ANOVA) (Figs. 2B and 2C). In 8 of the 9 patients who donated their brains, pathology stage as determined by in vivo ^18^F-flortaucipir PET was less than or equal to that determined postmortem (Fig. 3). In vivo staging and postmortem staging were not significantly correlated (Spearman *r*, 0.168; *P* = 0.67). Correlation analyses were also used to test the residuals of each staging variable (in vivo and postmortem staging) after regressing out clinical severity (PSPRS scores) and the interval from PET to time of death. The correlation was not statistically significant (Spearman *r*, 0.150; *P* = 0.70). Figure 4 shows examples of ^18^F-flortaucipir nondisplaceable binding potential maps and corresponding postmortem staining data for patients who were classified in stage II (patient 4) and stage IV (patient 26) by both in vivo staging and postmortem staging.

## DISCUSSION

The principal finding of the present study was that ^18^F-flortaucipir PET does not provide accurate in vivo staging corresponding to neuropathologic staging for PSP. The nominal stage derived from ^18^F-flortaucipir PET did not correlate with disease severity or relate to staging postmortem.

As a result of the data-driven in vivo staging system, compared with controls, we observed higher ^18^F-flortaucipir binding in the globus pallidus in all but 1 patient, with a few patients showing increased ^18^F-flortaucipir binding in the occipital cortex (Fig. 2A). This regional distribution of ^18^F-flortaucipir binding was in line with the pathologic description of PSP and with what was previously described for ^18^F-flortaucipir in PSP (*13**,**17**,**18**,**20*). Whereas the ^18^F-flortaucipir binding patterns allowed us to nominally apply PSP pathology staging in vivo, the in vivo staging was not systematically predictive of pathology staging postmortem. As expected because of the time interval between the PET scan and autopsy, in 8 of 9 cases with autopsy, the individual in vivo staging was less than or equal to the postmortem staging. However, 4 patients who were labeled as stage IV in vivo were then classified in 4 different stages postmortem (Fig. 3). Neither clinical severity nor the time interval between the PET scan and death was useful for predicting the individual postmortem stage from in vivo staging.

The number of patients with a positive signal for ^18^F-flortaucipir in the cerebellum (*n* = 29) exceeded the number of patients with a positive result for frontal ^18^F-flortaucipir binding (*n* = 10). Although this finding might reflect earlier involvement of the cerebellum in our cohort, regional differences in the density of tau aggregates and predominant cytopathologies could contribute to regional differences in tracer retention (*11**,**13**,**21*)—for example, neuronal and oligodendroglial tau predominates in the cerebellum, whereas astrocytic tau predominates in cortical regions.

Off-target binding for ^18^F-flortaucipir is well characterized, but this problem alone would still leave open the possibility of quantifying tau pathology in areas without significant monoamine oxidase levels or neuromelanin, such as the cerebellum and medial frontal cerebral cortex (*22*). However, recent PET-to-autopsy correlational studies suggested that ^18^F-flortaucipir PET does not reliably correspond to postmortem tau pathology in non-Alzheimer tauopathies (*13**,**23*). This finding suggests that ^18^F-flortaucipir lacks sensitivity in non-Alzheimer tau pathology. This characteristic might explain the underperformance of this tracer in defining an in vivo classification that systematically aligns with postmortem staging. Next-generation tau tracers might prove to be more useful for tracking in vivo PSP pathology progression because of a combination of good affinity for 4-repeat tau and lower off-target binding to monoamine oxidases (i.e., ^18^F-PI-2620 (*24*)). However, evidence from PET-to-autopsy studies for these new ligands is needed, together with better segmentation and signal detection from small regions. These features would be particularly important for early-stage pathology detection and the classification of stage I/II of the system of Kovacs et al. (*7*).

## CONCLUSION

We conclude that ^18^F-flortaucipir PET is not a useful marker of the neuropathologic stage in PSP, despite increased binding and some regional concordance between tau pathology and ligand binding. This analytic approach, seeking to mirror in vivo neuropathology staging with PET-to-autopsy correlational analyses, could be applied to test next-generation tau PET tracers. However, comparisons with postmortem data are also required.

## Notes

KEY POINTS **QUESTION:** Can the novel postmortem pathologic staging of PSP be reproduced in vivo with ^18^F-flortaucipir PET? **PERINENT FINDINGS:** Conditional inference tree analyses were performed on regional ^18^F-flortaucipir PET binding potential values to define in vivo staging in 42 patients with probable PSP, comparing the results in 9 participants with postmortem confirmation of the diagnosis and stage. ^18^F-flortaucipir PET did not provide accurate in vivo staging of PSP; in particular, the nominal stage derived from ^18^F-flortaucipir PET did not correlate with disease severity or relate to staging postmortem. **IMPLICATIONS FOR PATIENT CARE:** This analytic approach, seeking to mirror in vivo neuropathology staging with PET-to-autopsy correlational analyses, might be more effective with next-generation tau PET tracers.
