# Supplementary material for: In vivo 18F-flortaucipir PET does not accurately support the staging of progressive supranuclear palsy
Source: J Nucl Med. Author manuscript; Available in PMC 2022 Jul 4. (PMC7612961; doi:10.2967/jnumed.121.262985)
Supplement: Supplementary file 1 [file EMS140675-supplement-Supplemental_Materials.pdf]

SUPPLEMENTAL MATERIALS AND METHODS

Standard-deviation (SD) *in vivo* staging approach. First, binary severity scores were assigned using 1.5 SD from control mean as the threshold (w-score ≤ 1.5 SD = 0 or none; w-score > 1.5 SD = 1 or abnormal binding), and the same rules as for data-driven step 1 were used to assign each participant to stages I/II, III/IV or V/VI. Second, within each stage defined in the previous step, a 3-point pathology severity system was applied using 1.5 and 3 SD as thresholds across all regions (w-score ≤ 1.5 SD: 0 or none; w-score > 1.5 SD: 1 or mild/moderate; w-score > 3 SD: 2 or moderate/severe) and one of the six stages were assigned accordingly (stage I-VI). Analysis of variance (ANOVA) was applied to analyse differences among means of disease severity (PSPRS) between stages.

SUPPLEMENTAL RESULTS

***In vivo* staging based on standard-deviation thresholds.** The same set of decision rules used for data-driven staging applied to the standard-deviation approach identified N=12 patients in stage I/II, N=15 in stage III/IV, N=6 in stage V/VI, while N=9 were not classifiable. The explorative sub-staging identified N=5 patients in stage I, N=7 in stage II, N=10 in stage III, N=5 in stage IV and N=6 in stage V (Supplemental Figure 2A). Across all patients, *in vivo* stages did not significantly relate to clinical severity (ANOVA p>0.05, Supplemental Figure 2B and Supplemental Figure 2C). Applying the same approach on controls, N=36 participants were classified in no stage and N=3 in stage I. In 8 of the 9 patients who donated their brains, pathology stage as determined by *in vivo* ^{18}F-FTP PET, was less than or equal to that determined at *post-mortem* (Supplemental Figure 2D), while one patient was not classifiable with the standard-deviation staging approach.

SUPPLEMENTAL FIGURES

Supplemental Figure 1. Regions of interest considered for *in vivo* staging and corresponding regional w-scores. Panel A: Orthogonal planes through the regions of interest (ROIs) used to determine ^{18}F-FTP non-displaceable binding potential for *in vivo* staging overlaid on the native space T1 MRI for two representative patients. The ROIs are: globus pallidus (cyan); cerebellar white matter (yellow); dentate nucleus (red); middle frontal gyrus (blue); and lingual gyrus and cuneus (green). Panel B: Regional w-scores accounting for age and scanner type. For our analyses, cerebellar white matter (WM) was combined with dentate nucleus, and lingual gyrus was combined with the cuneus.

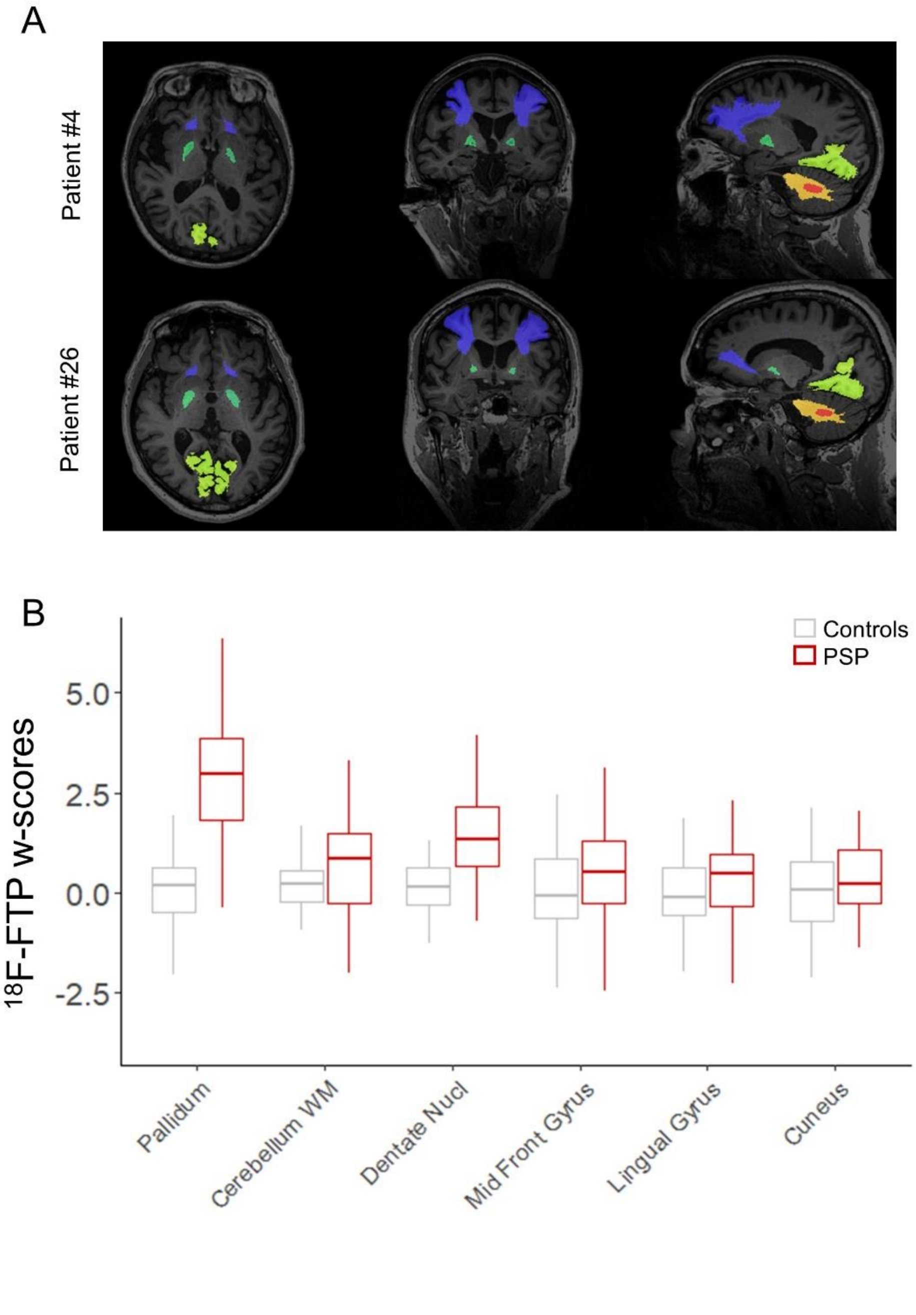

Supplemental Figure 2. *In vivo* staging based on standard-deviation thresholds. Panel A: severity scores are reported for each group of regions considered to define *in vivo* stages (STEP 1: 0 = absent; 1 = present) and sub-stages (STEP 2: 0 = none; 1 = mild/moderate pathology; 2 = moderate/severe pathology): progressive supranuclear palsy (PSP), PSP-Richardson's syndrome (-RS), PSP-frontal (-F), PSP-progressive gait freezing (-PGF), PSP-oculomotor (-OM), PSP-corticobasal syndrome (-CBS), globus pallidum (GP), cerebellum (CER, white matter and dentate nucleus), middle frontal gyrus (FR) and occipital lobe (OCC – lingual gyrus and cuneus). Panels B and C: boxplots of PSP rating scale (PSPRS) scores by stages defined with STEP 1 (panel B) and STEP 2 (panel C). Panel D: *in vivo* and *post-mortem* stages for 9 patients who underwent ^{18}F-FTP PET and donated their brains. For *in vivo* stages, results with both approaches are reported for the 9 patients.

A

	STEP 1					STEP 2				
Phenotype	GP	CER	FR	OCC	Stage	GP	CER	FR	OCC	Sub-Stage
PSP-RS	1	0	0	0	I/II	1	0	0	0	I
PSP-RS	1	0	0	0	I/II	1	0	0	0	I
PSP-RS	1	0	0	1	I/II	1	0	0	1	I
PSP-RS	1	0	0	0	I/II	1	0	0	0	I
PSP-RS	1	0	0	0	I/II	1	0	0	0	I
PSP-RS	1	0	0	0	I/II	2	0	0	0	II
PSP-RS	1	0	0	0	I/II	2	0	0	0	II
PSP-RS	1	0	0	0	I/II	2	0	0	0	II
PSP-F	1	0	0	0	I/II	2	0	0	0	II
PSP-PGF	1	0	0	0	I/II	2	0	0	0	II
PSP-RS	1	0	0	0	I/II	2	0	0	0	II
PSP-RS	1	0	0	0	I/II	2	0	0	0	II
PSP-RS	1	1	1	0	III/IV	1	1	1	0	III
PSP-RS	1	1	0	0	III/IV	1	1	0	0	III
PSP-RS	1	1	0	0	III/IV	1	1	0	0	III
PSP-RS	1	1	0	0	III/IV	1	1	0	0	III
PSP-RS	1	1	0	0	III/IV	2	1	0	0	III
PSP-RS	1	1	0	0	III/IV	2	1	0	0	III
PSP-RS	1	1	0	0	III/IV	2	1	0	0	III
PSP-F	1	1	0	0	III/IV	2	1	0	0	III
PSP-RS	1	1	0	0	III/IV	2	1	0	0	III
PSP-OM	1	1	0	0	III/IV	2	1	0	0	III
PSP-RS	1	1	0	0	III/IV	1	2	0	0	IV
PSP-RS	1	0	1	0	III/IV	1	0	2	0	IV
PSP-RS	1	1	1	0	III/IV	2	2	1	0	IV
PSP-F	1	1	1	0	III/IV	2	2	1	0	IV
PSP-F	1	0	1	0	III/IV	2	0	2	0	IV
PSP-RS	1	1	0	1	V/VI	1	2	0	1	V
PSP-RS	1	1	1	1	V/VI	2	2	1	1	V
PSP-CBS	1	1	1	1	V/VI	2	1	1	1	V
PSP-RS	1	1	1	1	V/VI	2	1	1	1	V
PSP-RS	1	1	1	1	V/VI	2	1	1	1	V
PSP-RS	1	0	1	1	V/VI	2	0	2	1	V
PSP-RS	0	0	0	0	NULL	0	0	0	0	NULL
PSP-RS	0	1	0	0	NULL	0	1	0	0	NULL
PSP-RS	0	0	0	0	NULL	0	0	0	0	NULL
PSP-RS	0	0	0	0	NULL	0	0	0	0	NULL
PSP-RS	0	0	0	0	NULL	0	0	0	0	NULL
PSP-RS	0	0	0	0	NULL	0	0	0	0	NULL
PSP-RS	0	0	0	0	NULL	0	0	0	0	NULL
PSP-RS	0	1	0	0	NULL	0	1	0	0	NULL
PSP-RS	0	0	0	0	NULL	0	0	0	0	NULL

B

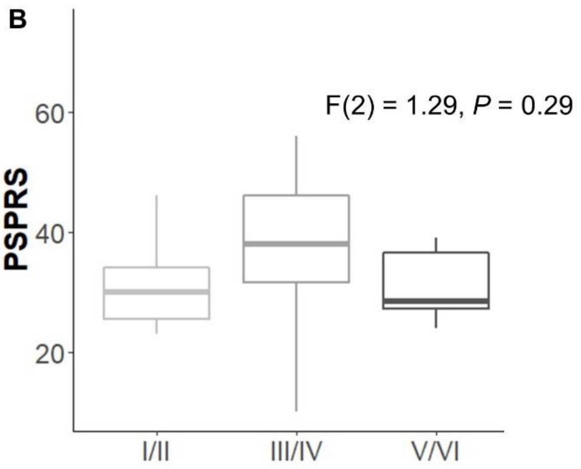

C

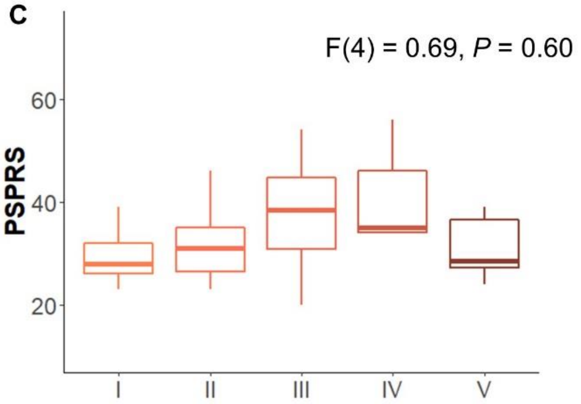

D

Syndrome	In vivo stage: data-driven	In vivo stage: std-dev	Post mortem stage
PSP-RS	I	NULL	V
PSP-RS	II	II	II
PSP-RS	III	II	III
PSP-RS	IV	III	III
PSP-RS	IV	III	IV
PSP-RS	IV	III	IV
PSP-RS	IV	IV	V
PSP-RS	IV	III	V
PSP-F	IV	IV	VI
